# Supplementary material for: Reconstitution of dynein transport to the microtubule plus end by kinesin
Source: eLife. 2014 Jun 10;3:e02641. doi: 10.7554/eLife.02641 (PMC4046564; doi:10.7554/eLife.02641)
Supplement: Supplementary file 1. — ImageJ macro used to calculate motor velocities and run lengths from kymographs. DOI: http://dx.doi.org/10.7554/eLife.02641.017 [file elife02641s001.docx]

**CODE**

**Custom Image J macro used to calculate motor velocities and run lengths from kymographs.**

//Automated Calculation of Velocity and Run lengths

//This function requires the use of ROI Manager, and will iterate all the ROIs

//To retrieve information of velocity and run lengths

//Press Shift+K to call the macro

//Weihong Qiu, Harvard Medical School, 03/27/2009

// Last update: 08/09/2010

macro "KymographSegLine [K]" {

nRoiCount = roiManager("count");

if(nRoiCount==0) {

exit("Empty ROI Manager!");

}

kymograph=getString("Select Kymograph","Kymograph.tif");

if(isOpen(kymograph)==true)

selectWindow(kymograph);

else

exit("Please check your kymograph!");

for(index =0; index<nRoiCount;index++) {

roiManager("select", index);

getSelectionCoordinates(x, y);

sum_dx=0;

sum_dy=0;

row = nResults;

for (i=0; i<x.length-1; i++){

dx_now=abs(x[i+1]-x[i]);

sum_dx=sum_dx+dx_now;

dy_now=abs(y[i+1]-y[i]);

if (dy_now==0)dy_now=1;

sum_dy=sum_dy+dy_now;

}

setResult("Total Displacement", row, sum_dx);

setResult("Total Time", row, sum_dy);

setResult("Average speed", row, (sum_dx/sum_dy));

}

updateResults();

}

}
